# Draft Genome Sequences of *Helicobacter pylori* Strains Isolated from Regions of Low and High Gastric Cancer Risk in Colombia

**DOI:** 10.1128/genomeA.00736-13

**Published:** 2013-09-19

**Authors:** Alexander Sheh, M. Blanca Piazuelo, Keith T. Wilson, Pelayo Correa, James G. Fox

**Affiliations:** Division of Comparative Medicine, Massachusetts Institute of Technology, Cambridge, Massachusetts, USAa; Division of Gastroenterology, Department of Medicine, Vanderbilt University School of Medicine, Nashville, Tennessee, USAb; Department of Cancer Biology, Vanderbilt University School of Medicine, Nashville, Tennessee, USAc; Department of Pathology, Microbiology and Immunology, Vanderbilt University School of Medicine, Nashville, Tennessee, USAd; Veterans Affairs Tennessee Valley Healthcare System, Nashville, Tennessee, USAe; Department of Biological Engineering, Massachusetts Institute of Technology, Cambridge, Massachusetts, USAf

## Abstract

The draft genome sequences of six Colombian *Helicobacter pylori* strains are presented. These strains were isolated from patients from regions of high and low gastric cancer risk in Colombia and were characterized by multilocus sequence typing. The data provide insights into differences between *H. pylori* strains of different phylogeographic origins.

## GENOME ANNOUNCEMENT

*Helicobacter pylori* is a Gram-negative, microaerophilic, helix-shaped bacterium that colonizes the stomachs of at least half of the world’s human population (1). In most cases, *H. pylori* can persist in the human stomach without health consequences, though it is a risk factor for chronic gastritis, gastric or duodenal ulcers, and gastric cancer (2).

In the present report, we announce the genome sequencing of six strains of *Helicobacter pylori*. These strains were isolated from three patients from the low-gastric-cancer-risk region of Tumaco, Colombia (strains PZ5004, PZ5024, and PZ5026), and three patients from the high-gastric-cancer-risk region of Pasto, Colombia (strains PZ5056, PZ5080, and PZ5086). These strains have been previously classified by multilocus sequence typing (MLST) and found to be of African (PZ5004 and PZ5024) and of European (PZ5026, PZ5056, PZ5080, and PZ5086) origins (3). Microarray analysis of these six strains demonstrated that clustering of transcriptomes also sorted strains based on their phylogeographic origins, with greater expression of virulence genes, such as *cagA* and *vacA*, in European strains (4). European strains also induced greater interleukin 8 (IL-8) expression while reducing apoptosis (4). The genome data provide insights about the genomic diversity of *H. pylori* from two sites with different incidence risks for gastric cancer and may help determine the underlying causes for differential transcription and virulence. The RNase-treated DNAs from the six isolates were sequenced using an Illumina MiSeq system as described previously (4). The 150-bp paired-end sequencing reads generated by MiSeq were assembled into contigs using Velvet (5). Sequences were annotated using the NCBI Prokaryotic Genomes Automatic Annotation Pipeline (PGAAP) (6). Pertinent statistics are summarized in Table 1. Overall, average GC contents of 38.65% and 1,660 coding DNA sequences (CDS) were found. The median coverage depth ranged from 128- to 246-fold.

**TABLE 1:** Pertinent statistics for sequenced Colombian strains

Strain	NCBI accession number	Genome length (bp) (Velvet)	No. of contigs (Velvet)	Median coverage depth (*n*-fold) (Velvet)	GC content (%) (Velvet)	No. of genes (PGAAP)	No. of CDS (PGAAP)
PZ5004	ASZF00000000	1,569,902	303	246	38.75	1,727	1,662
PZ5024	ASYS00000000	1,496,849	413	145	38.31	1,706	1,643
PZ5026	ASYT00000000	1,604,992	253	212	38.73	1,731	1,668
PZ5056	ASYU00000000	1,578,164	335	128	38.72	1,753	1,695
PZ5080	ASYV00000000	1,597,127	283	194	38.62	1,738	1,677
PZ5086	ASYW00000000	1,547,845	295	207	38.76	1,672	1,616

### Nucleotide sequence accession numbers.

The genome sequences of *Helicobacter pylori* strains PZ5004, PZ5024, PZ5026, PZ5056, PZ5080, and PZ5086 were deposited at GenBank with the accession numbers listed in Table 1.
